# Translation Imaging in Parkinson’s Disease: Focus on Neuroinflammation

**DOI:** 10.3389/fnagi.2020.00152

**Published:** 2020-06-05

**Authors:** Sara Belloli, Michele Morari, Valentina Murtaj, Silvia Valtorta, Rosa Maria Moresco, Maria Carla Gilardi

**Affiliations:** ^1^Institute of Molecular Bioimaging and Physiology (IBFM), CNR, Milan, Italy; ^2^Nuclear Medicine Department, San Raffaele Scientific Institute (IRCCS), Milan, Italy; ^3^Section of Pharmacology, Department of Medical Sciences, National Institute for Neuroscience, University of Ferrara, Ferrara, Italy; ^4^PhD Program in Neuroscience, School of Medicine and Surgery, University of Milano-Bicocca, Milan, Italy; ^5^Medicine and Surgery Department, University of Milano-Bicocca, Milan, Italy

**Keywords:** Parkinson’s disease, early disease markers, neuroinflammation, oxidative stress, PET radioligands, PD models, microglia

## Abstract

Parkinson’s disease (PD) is characterized by the loss of dopaminergic neurons in the substantia nigra pars compacta (SNpc) and the appearance of α-synuclein insoluble aggregates known as Lewy bodies. Neurodegeneration is accompanied by neuroinflammation mediated by cytokines and chemokines produced by the activated microglia. Several studies demonstrated that such an inflammatory process is an early event, and contributes to oxidative stress and mitochondrial dysfunctions. α-synuclein fibrillization and aggregation activate microglia and contribute to disease onset and progression. Mutations in different genes exacerbate the inflammatory phenotype in the monogenic compared to sporadic forms of PD. Positron Emission Tomography (PET) and Single Photon Emission Computed Tomography (SPECT) with selected radiopharmaceuticals allow *in vivo* imaging of molecular modifications in the brain of living subjects. Several publications showed a reduction of dopaminergic terminals and dopamine (DA) content in the basal ganglia, starting from the early stages of the disease. Moreover, non-dopaminergic neuronal pathways are also affected, as shown by *in vivo* studies with serotonergic and glutamatergic radiotracers. The role played by the immune system during illness progression could be investigated with PET ligands that target the microglia/macrophage Translocator protein (TSPO) receptor. These agents have been used in PD patients and rodent models, although often without attempting correlations with other molecular or functional parameters. For example, neurodegeneration and brain plasticity can be monitored using the metabolic marker 2-Deoxy-2-[^18^F]fluoroglucose ([^18^F]-FDG), while oxidative stress can be probed using the copper-labeled diacetyl-bis(N-methyl-thiosemicarbazone) ([Cu]-ATSM) radioligand, whose striatal-specific binding ratio in PD patients seems to correlate with a disease rating scale and motor scores. Also, structural and functional modifications during disease progression may be evaluated by Magnetic Resonance Imaging (MRI), using different parameters as iron content or cerebral volume. In this review article, we propose an overview of *in vivo* clinical and non-clinical imaging research on neuroinflammation as an emerging marker of early PD. We also discuss how multimodal-imaging approaches could provide more insights into the role of the inflammatory process and related events in PD development.

## Introduction: Parkinson’s Disease Pathology

Parkinson’s disease (PD) is a neurodegenerative disorder characterized by the progressive loss of dopaminergic neurons in substantia nigra (SN) pars compacta (SNpc), and the appearance of α-synuclein (α-syn) positive aggregates known as Lewy bodies (LBs). PD is a multisystemic disorder since Lewy pathology also affects nondopaminergic areas (Braak and Del Tredici, 2017; Giguere et al., 2018). PD is clinically characterized by cardinal motor symptoms (hypokinesia, bradykinesia, tremor, rigidity, postural instability) which are accompanied by a plethora of non-motor symptoms affecting cognition, autonomic and sensory functions, mood and sleep (Poewe et al., 2017). Motor symptoms essentially derive from the degeneration of SNpc neurons and the accompanying dysfunctions in brain regions and neurostructural pathways involved in the cortico-basal ganglia-thalamocortical loop, whereas non-motor symptoms are due to degeneration of non-dopaminergic areas. The prevalence of PD increases with age (from 41/100,000 individuals between 40–49 years up to 1,903/100,000 individuals >80 years), and is gender-dependent, being twice more common in males than females (Van Den Eeden et al., 2003; Pringsheim et al., 2014). The incidence of PD is progressively increasing worldwide, and the number of people with PD is expected to exceed 12 million by 2040, which has brought some authors to label PD as a pandemic (Dorsey and Bloem, 2018; Dorsey et al., 2018). Most PD cases are classified as idiopathic and only <10% have a clear monogenic origin (familial PD). The etiology of idiopathic PD remains yet unknown although genetic and environmental factors, and their interaction, contribute to the onset and progression of the disease. Genome-wide studies have identified 90 common variants associated with the risk of PD (Keller et al., 2012; Nalls et al., 2019), and the genetic contribution to clinical heterogeneity and progression of PD is also under investigation (Iwaki et al., 2019). A significant risk association was found with variants of Human Leukocyte Antigen–Major Histocompatibility Complex II (HLA–MHC II) genes (Hamza et al., 2010; Pierce and Coetzee, 2017), Nitric Oxide Synthase 1 and 2a (NOS1 and NOS2A) genes (coding for neuronal and inducible isoforms of NOS, respectively (Hancock et al., 2008), Tumor Necrosis Factor-α (TNF-α) and its Receptor 1 (TNFR1) genes (coding for the proinflammatory cytokine and its receptor; Krüger et al., 2000), suggesting a role of neuroinflammation in PD etiology (Tansey et al., 2007; Hirsch and Hunot, 2009; Johnson et al., 2019). To further strengthen this view, an association between polymorphisms of the gene encoding for the proinflammatory cytokine IL-1β and PD was found in different ethnic backgrounds (Mattila et al., 2002; McGeer and McGeer, 2002; Schulte et al., 2002). Also, other studies found a correlation between polymorphisms of the genes of IL-1β (Nishimura et al., 2000), TNF (Nishimura et al., 2001) and IL-10 (an anti-inflammatory cytokine; Håkansson et al., 2005) with age at onset of PD.

Several studies showed that environmental factors act to increase (pesticides, solvents, head trauma, stress) or reduce (caffeine, smoking, diet, physical exercise) the risk of PD (Ascherio and Schwarzschild, 2016; Marras et al., 2019). Some environmental toxins associated with PD induce microglial activation in experimental animals (see for reviews Cebrián et al., 2015; Joers et al., 2017) and PD patients (Teismann et al., 2003). Among these, 1-methyl-4-phenyl-1,2,3,6-tetrahydropyridine (MPTP; Członkowska et al., 1996; Wu et al., 2002; Sugama et al., 2003; Campos-Acuña et al., 2019), the pesticide rotenone (Sherer et al., 2003; Emmrich et al., 2013; Gao et al., 2013) and the herbicide paraquat (Cicchetti et al., 2005; Purisai et al., 2007). Some epidemiological studies pointed out that the use of some nonsteroidal anti-inflammatory drugs (NSAIDs) is associated with a lower risk of PD (Chen et al., 2003, 2005; Wahner et al., 2007; Gagne and Power, 2010; Rees et al., 2011); other studies failed to prove an association (Ton et al., 2006; Hancock et al., 2007, 2008; Becker et al., 2011; Driver et al., 2011). The concept needs further exploration as NSAIDs have the potential for serious adverse cardiovascular events such as stroke (Caughey et al., 2011). Nonetheless, in addition to MPTP and rotenone, another parkinsonian toxin used to induce PD in rats and mice, i.e., 6-hydroxydopamine (6-OHDA), causes microglial activation and *in vivo* dopamine (DA) cell loss (Akiyama and McGeer, 1989; Cicchetti et al., 2002; Depino et al., 2003). Peripheral inflammation is also thought to play a role in PD etiology (Johnson et al., 2019), as proinflammatory compounds such as lipopolysaccharides (LPS) not only induced *per se* the loss of midbrain neurons (Herrera et al., 2000; Arai et al., 2004) but also potentiated the neurodegeneration induced by MPTP, rotenone, and 6-OHDA (Gao et al., 2003; Koprich et al., 2008; Pott Godoy et al., 2008). More recently, the role of gut microbiota in PD etiology has emerged (Scheperjans et al., 2015; Breen et al., 2019). Interestingly, the gut microbiota is shown to influence microglia activity in the brain through the production of short-chain fatty acids (Erny et al., 2015), and gut microbiota complexity is reported to enhance microglia activation and α-syn-induced pathology in a mouse model of PD (Sampson et al., 2016).

Although it has been established that neuroinflammation is coupled to neurodegeneration of the monoaminergic system in PD, it is still unclear if the activation of the immune system is a cause or consequence of DA cell loss. The observation that at the early pre-symptomatic stage, α-syn aggregation can promote microglia activation and neuronal dysfunction without cell death would suggest that neuroinflammation is not only a consequence of neurodegeneration (Sanchez-Guajardo et al., 2013). Also consistent with the view that the inflammatory response is instrumental to experimentally-induced neurodegeneration, blockade of microglial activation and the inflammatory response attenuates DA neuron loss in different animal models of PD (Wu et al., 2002; Sánchez-Pernaute et al., 2004; Pott Godoy et al., 2008; Tentillier et al., 2016).

The discovery that α-syn is associated with familiar forms of PD (Polymeropoulos et al., 1997) and is the main component of LBs (Spillantini et al., 1997) has shed new light on the pathogenesis of the disease and the contribution of neuroinflammation. It is now accepted that that α-syn misfolding and aggregation are key events in PD pathogenesis in both familial and sporadic forms. α-syn localizes in different cell compartments but is most abundant in the presynaptic terminal (Iwai et al., 1995) where it regulates vesicular biogenesis, trafficking, and synaptic transmission (for a recent review see Sulzer and Edwards, 2019). Misfolded and/or aggregated α-syn triggers a cascade of events, among which microglia activation, that lead to dopaminergic neurons demise. α-syn released from neurons in the extracellular space has been shown to directly activate microglia through the Toll-like receptor 2 (TLR2; Kim et al., 2013). α-syn can also be taken up by microglial cells (Zhang et al., 2005). This causes microglial activation with the release of reactive oxygen species (ROS) and inflammatory products leading to DA neuron death.

Transgenic mice overexpressing human wild-type α-syn under the tyrosine hydroxylase (Su et al., 2008) or the Thy1 (Watson et al., 2012) promoters, showed an increase of the activated microglia and expression of TNF-α in the striatum and SNpc. Interestingly, temporal analysis of the microglia responses revealed that the striatum was affected earlier than SNpc. Moreover, also the delivery of the A53T pathogenic variant of α-syn using an adeno-associated virus serotype 2 (AAV2) in the rat SN induced striatal inflammation and disrupted nigro-striatal axon morphology and function before nigral cell loss (Chung et al., 2009). Likewise, AAV2-mediated overexpression of human wild-type α-syn in mice activated microglia, upregulated Nuclear Factor Kappa-light-chain-enhancer of activated B cells (NF-κb) signal and promoted neuronal death through Fc gamma receptors (FcγR) activation, suggesting a pathogenic role for humoral immunity in this model (Cao et al., 2010). Finally, the inflammatory response has been investigated in the most recent model of PD where preformed fibrils (PFF) of α-syn are injected into the striatum to cause endogenous α-syn aggregation and spreading in the brain (Luk et al., 2012). Different studies both in α-syn transgenic overexpressors (Theodore et al., 2008; Watson et al., 2012) and non-transgenic (Duffy et al., 2018; Earls et al., 2019) rodents pointed out an increased microglia activation in SNpc that precedes neuronal cell loss.

All these findings strongly indicate that neuroinflammation and in particular, microglia activation, is linked to α-syn pathology, playing a key role in PD pathogenesis.

## Inflammation in Parkinson’s Disease

As for other neurodegenerative disorders, inflammation in the brain and periphery represents a hallmark of PD. Microglia are resident cells of the brain immune system that mediate inflammatory response or trophic function depending on the environment surrounding cells. However, the precise role of microglia and peripheral myeloid cells in PD is not clear. Microglia consist of 10% of brain cells. Resting microglia sense the presence of pathogens, trauma, oxidative stress, neuronal damages, and modify their morphology and functions migrating to the damaged region where also peripheral monocytes are recruited. Different activated microglial phenotypes coexist and can be classified, in partial analogy to peripheral macrophage phenotypes, in M1, also referred to as the classical or proinflammatory phenotype, and in the alternative M2 group, which comprises the M2a, M2b and M2c phenotypes. M1 macrophages react to injury releasing proinflammatory cytokines (Walker and Lue, 2015) and then could return to a resting state. M2a macrophages are induced by IL-4 and IL-13 and release neurotrophic factors and IL-10, which exert an anti-inflammatory role. The other M2 phenotypes, M2b and M2c are denominated type II or regulatory and deactivated macrophages, respectively. M2b macrophages can be polarized by several stimulatory factors, which include cytokines (IL-4, IL-10, and IL-13), glucocorticoids, and IC (Boche et al., 2013). In addition to proinflammatory cytokines (IL-1β, IL-6, and TNF-β), M2b cells also express and secrete substantial amounts of the anti-inflammatory cytokine IL-10 and low levels of IL-12. Finally, M2c macrophages are induced by IL-10 *via* activating Signal Transducer and Activator of Transcription 3 (STAT3) through the IL-10 receptor, and strongly exhibit anti-inflammatory activities by releasing large amounts of IL-10 and pro-fibrotic activity by secreting high levels of Transforming Growth Factor β (TGF-β; Martinez and Gordon, 2014; Wang et al., 2019). Despite several studies are focused on this issue, the specific role and enrichment conditions of the various phenotypes described are not completely elucidated (Cherry et al., 2014). However, the modulation of microglial phenotypes has been proposed as a potential therapeutic target for PD (Subramaniam and Federoff, 2017). In terms of morphology, microglia are classified in different types: (i) resting, that promote synaptic pruning, neuronal plasticity and exert surveillance, characterized by a ramified shape; (ii) amoeboid, that react to immune stimuli and stress (de Pablos et al., 2014), promote inflammation (George et al., 2019), motility and phagocytosis, with increased and longer ramifications; and (iii) dark, that interact with neurons and synapses, participate in neuronal remodeling (Bisht et al., 2016), increase inflammation and release nitric oxide (Lecours et al., 2018), with short and twisted processes and phagocytic inclusions. All these types, that often coexist, have been described or associated with PD. During aging, microglia become senescent and dysfunctional (microglial response is poorly correlated with neurodegeneration following chronic, low-dose MPTP administration in monkeys; Hurley et al., 2003). In PD models and PD patients, microglial cells show MHC class II marker (McGeer et al., 1988; Imamura et al., 2003), display a ramified morphology and express CD11a, CD68, TNF-α, and IL-6. At the molecular level, increased production of IL-1β, IL-2, IL-4, IL-6, TNF-α, TGF-α, and TGF-β1 is also reported (Nagatsu et al., 2000). Some of these markers are common to the so-called primed microglia, typically during aging. Therefore, aging and other factors might promote neuroinflammation and neurodegeneration. However, PD is not limited to aging, although it represents its main risk factor. Indeed, the severity of nigrostriatal damage in the early phase of sporadic PD is not dependent on age at onset as indicated by Panzacchi et al. (2008) measuring the expression of DA transporter (DAT) in early phase young and old PD patients. Microglia promote neuronal survival, pruning, and dendritic spine formation through the release of neurotrophic factors (Wang et al., 2019). In PD patients and mice exposed to MPTP or 6-OHDA, nigral levels of Brain-Derived Neurotrophic Factor (BDNF) are reduced and this may be related to impaired function of microglial cells (Nagatsu and Sawada, 2005). Another relevant point is that a huge number of genes associated with PD are expressed in microglia and astrocytes. Protein Deglycase DJ-1 (Trudler et al., 2014), PTEN-Induced Kinase 1 (PINK1), Leucine-Rich Repeat Kinase 2 (LRRK2; Ho et al., 2018), Triggering Receptor Expressed on Myeloid Cells 2 (TREM2; Zhang et al., 2018) and α-syn regulate sensing, the inflammatory response of microglia and phagocytosis, and modification in their activity might influence the ability of microglial cells to repair an injury or sustain neuron viability and functions (see for a review Ferreira and Romero-Ramos, 2018).

Moreover, most of the genes involved in PD such as DJ-1, α-syn, Ca^2+^-independent phospholipases A2 (iPLA_2_), ATPase cation transporting 13A2 (ATP13A2), PINK1, and parkin have a regulatory function on astrocytes. As recently reviewed by Booth et al. (2017), these genes participate in the control of astrocytes activation during neuroinflammation, lipid metabolism and arachidonic acid release, mitochondrial and lysosomal function. α-syn staining has been found in the astrocytes of several brain regions in PD patients, including the thalamus and cerebral cortex. According to Booth et al. (2017), astrocytes dysfunction participates to neuronal toxicity through a reduction in water transport and neurotrophic factor release, and an increase in inflammatory signaling *via* TLR4, Interferon-gamma (IFN-γ), and NOD-, LRR- and pyrin domain-containing protein 3 (NLPR3) inflammasome pathway. An increase in its activity is associated with reduced glutamate buffering and α-syn processing (Booth et al., 2017).

Within the immune system, α-syn seems to play a critical role. MHC class II expressing microglia is associated with α-syn deposition (Croisier et al., 2005) and PD patients show T-cells response against α-syn peptides, indicating that immune system tries to respond to neuronal events related to α-syn (Sulzer et al., 2017).

Interestingly, microglial cells display region-specific transcriptome profiles as revealed by post-mortem analysis in mice and patients (Grabert et al., 2016), with regions expressing the transcript more related to synaptic plasticity and neurogenesis like the hippocampus, and others to cell metabolism and metabolic functions. Using laser capture analysis, Mastroeni et al. (2018) showed a higher number of modified transcripts in the SN of PD patients than in the hippocampus, and these transcripts were related to synaptic transmission.

As previously stated, another population of cells potentially involved in PD is represented by astrocytes. Reactive astrocytosis characterized by change of morphology and release of chemokines is common during neurodegenerative processes. Contrary to other neurodegenerative disorders, no astrocytosis or even a reduction in the astrocyte content have been described post-mortem in PD patients (Cook et al., 2016). However, astrocytes exert several functions in the central nervous system (CNS) including structural, metabolic activity, synaptic regulation, production and release of neurotrophic molecules, blood-brain barrier (BBB) regulation, and protection from neurotoxins.

Finally, regarding inflammation and PD, several reports are focused on peripheral cells or peripheral inflammatory markers. Extensive coverage of these studies is beyond the focus of this review article. However, from these studies, deregulation of immune system activity emerged: (a) increased levels of inflammatory markers such as TNF and TNF-1 receptor; (b) association between circulating IL-6 levels and PD, modifications in monocytes and lymphocytes activity and concentration; (c) increased susceptibility to infectious or inflammatory disease including gut disorders; and finally (d) presence of infiltrated cytotoxic T lymphocytes (CD4+ and CD8+) in SN (Brochard et al., 2009). The peripheral immune system has been shown to infiltrate the SN of PD patients, suggesting a potential BBB dysfunction in PD patients (Hasegawa et al., 2000; Chen et al., 2008; Dufek et al., 2009; Devos et al., 2013).

## Imaging of Parkinson’s Disease

*In vivo* molecular imaging techniques such as Positron Emission tomography (PET) and Single Photon Emission Computed Tomography (SPECT) provide relevant information for the understanding of the pathophysiology of PD, particularly using radiopharmaceuticals that specifically target Dopamine (DA) nerve endings or cell bodies (Weingarten et al., 2015; Meles et al., 2017; Wang et al., 2017). Radiopharmaceuticals that target DAT, aromatic amino acid decarboxylase ([^18^F]-FDOPA; Brooks et al., 2003), or monoaminergic nerve ending vesicles (VMAT2; [^18^F]-Dihydrotetrabenazine; Frey et al., 1996; Hsiao et al., 2014), showed a marked binding reduction in different striatal and extra-striatal regions, already at the beginning of the symptomatic phase. [^123^I]-Ioflupane (DATScan^®^) is an approved SPECT ligand specific for DAT and represents a highly sensitive tool for patients with suspected Parkinsonian syndrome. In the clinical practice, DATScan^®^ is used for the differential diagnosis between essential tremor and PD, or between Lewy Body Dementia (LBD), Parkinson Dementia Disease (PDD) and PD (Brooks, 2016), when motor signs are fully evident and striatal neurodegeneration reaches 70%. The development of new generation PET systems and the validation of new ligands specific for different markers of monoaminergic pathways have improved the sensitivity of PD diagnosis without influencing patient’s management and survival (Liu et al., 2018). Dopaminergic neurons are not the only population affected in PD, as also clearly indicated *in vivo* by radiopharmaceuticals that target serotonergic or cholinergic neurons (Marcone et al., 2012). Radiopharmaceuticals that bind to the serotonin transporter (SERT), such as [^11^C]-(3-amino-4-(2-dimethylamino-methyl-phenyl-sulfonyl)-Benzonitrile) ([^11^C]-DASB), or to Acetylcholinesterase (AChE) enzyme as [^11^C]-methyl-4-piperidyl acetate ([^11^C]-MP4A), allow to map brain SERT in patients with different degrees of PD severity, or cholinergic denervation associated with progressive cognitive decline and loss of odor discrimination (Politis et al., 2010).

Despite the growing knowledge of PD risk factors and biomarkers, and progressive refinements in imaging techniques and preclinical studies on peripheral cells or neurons, the early molecular events that trigger PD onset remain poorly understood. Indeed, several studies now focus on the identification of preclinical molecular events in the early stages. As previously stated, the hypothesis that neuron damage starts from nerve endings and that synaptic impairment represents an early and key event in PD pathogenesis is also supported by *in vivo* PET studies. This issue has been investigated in different genetic mouse models, clearly showing that different mutations in genes such as SNCA or LRRK2 impact cortico-striatal synaptic transmission and plasticity, before neuronal death appears (Schirinzi et al., 2016).

Among the potential preclinical key events leading to PD, is the abnormal activation of brain immune system, or neuroinflammation (Hirsch and Hunot, 2009) that is considered a potential target for the development of novel therapeutic strategies.

## PET Imaging of Neuroinflammation

Neuroinflammation plays a critical role in the development and progression of different neurodegenerative disorders including PD (Infante-Duarte et al., 2008). Activation of the brain immune system involves both microglia and astrocytes although the specific cell phenotype at onset and during more advanced phases of PD is not well known (Hunot and Hirsch, 2003). The presence of activated microglia was first described by McGeer et al. (1988) in post mortem SN samples of PD patients and then confirmed by Banati et al. (1998). As previously stated, *in vitro* studies on microglia activation in animal models or autoptic samples from PD brains highlighted that microglia activation occurs in the early phase, before neuronal death, and plays different, although still not clearly identified roles during the course of the disease (Doorn et al., 2014; Hirsch and Hunot, 2009; Nissen et al., 2019).

PET radiopharmaceuticals that specifically bind the 18-KDa translocator protein (TSPO) enable to *in vivo* image the presence of activated microglial cell clusters and thus have the potential utility to monitor their levels and distribution during disease progression in living subjects (Politis et al., 2012). Unfortunately, PET studies with TSPO radiopharmaceuticals have been performed in small populations of PD patients and only a few of them compared the regional inflammatory response with the integrity of dopaminergic neurons. Ouchi et al. (2005) reported a parallel increase in microglial activation and dopaminergic terminal loss in the affected nigrostriatal pathway in early PD, as measured with the DAT radiopharmaceutical [^11^C]-2-β-carbomethoxy-3β-(4-fluorophenyl) tropane [^11^C]-CFT. According to these findings, the authors suggested that microglial cells contributed to neuronal degeneration well before symptoms appearance (Ouchi et al., 2005). Similar results were described by Gerhard et al. (2006) in the 2-year study, where they observed that TSPO levels remained stable over time and did not correlate with clinical signs or putaminal [^18^F]-FDOPA uptake. Based on these findings, other studies tried to verify if drugs able to modulate microglial cells, like minocycline and celecoxib, exerted a neuroprotective effect in a patient with PD or parkinsonism, but the results obtained were not conclusive (Bartels et al., 2010; Dodel et al., 2010). Using [^11^C]-acetamide, N-((2-(methoxy^−11^C)-phenyl)methyl)-N-(6-phenoxy-3pyridinyl)acetamide ([^11^C]PBR28) as radioligands, a reduction of TSPO binding was reported in the striatum and SN of PD patients treated for 8 weeks with Verdiperstat, an orally active irreversible myeloperoxidase inhibitor (Jucaite et al., 2015). The results suggested that the enzyme is involved in the regulation of microglial activation and thus it could be of potential interest as a target for PD treatment. Unfortunately, no other report confirmed these data and the link between the reduction of TSPO levels and positive clinical outcomes.

Using 1-(2-Chlorophenyl)-N-[^11^C]methyl-N-(1-methylpropyl)-3-isoquinoline carboxamide [^11^C]-PK11195, Iannaccone et al. (2013) showed the presence of clusters of activated microglial cells in nigro-striatal regions of early PD patients. The different distribution in nigro-striatal and cortical areas of these patients when compared to early-stage patients with LBD, indicates that cortical spreading follows a disease-specific topographical pattern involving the frontal aspects in PD and the occipital cortex in LBD. Also, Terada et al. (2016) using the second-generation TSPO-specific radioligand [^11^C]-N, N-diethyl-2-[2-(4-methoxyphenyl)-5,7-dimethylpyrazolo[1,5-a]pyrimidin-3-yl]acetamide ([^11^C]-DPA713) in early PD patients observed microglial activation all over the cortex. The [^11^C]-DPA713 PET repeated 1 year later showed an increased signal, especially in the temporal and occipital cortex. Overall, PET studies, although based on a limited sample of patients, showed increased binding to TSPO receptors, already at the beginning of symptoms and involving also cortical regions.

TSPO levels were imaged also in PD patients with cognitive decline. In PD patients with dementia, increased binding of [^11^C]-PK11195 was reported in different cortical regions including temporal, parietal and occipital cortex, and the observed increase was associated with cognitive impairment (Edison et al., 2013), as later confirmed by Fan et al. (2015).

In contrast to that previously reported, Varnas et al. (2019) reported no differences in TSPO ligand uptake in the brain of PD patients compared to controls, using [^11^C]-PBR28 as radioligand. However, this study was based on a small population of patients with PD and applied an isoform-sensitive tracer thus increasing the noise of the images obtained. A single-nucleotide polymorphism (rs6971) in exon 4 of the TSPO gene influences the binding profile of most of the second-generation TSPO radioligands leading to different binding levels between subjects carrying high-affinity phenotype, or mixed-affinity genotype (Owen et al., 2011).

As stated, TSPO is expressed in different eukaryotic cells of the myeloid lineage, and its expression increases in phagocytic cells. Preclinical studies showed that drugs modulating TSPO binding can protect from neurodegeneration, possibly through the modulation of ROS production and metabolism during microglia activation (Leaver et al., 2012; Gong et al., 2019).

A recent study performed both *in vitro* and *in vivo* using [^18^F]-DPA-713 and FACS showed that TNF but not IL-4 administration upregulated TSPO binding, suggesting that PET signal is not associated with an IL-4 associated microglia population (Pannell et al., 2020). However, it has been demonstrated that TSPO expression in human myeloid cells is negatively modulated by pro-inflammatory stimuli able to increase its level in murine cells (Owen et al., 2017). With the limitations of *in vitro* studies in immune cells, this finding suggests that the signal observed in patients might be more related to cell recruitment at the lesion site than to simple up-regulation of the receptors or modification in binding affinity due to conformational changes of the complex.

As previously stated, astrocytosis represents an intriguing target for imaging. Astrocytes number increases during aging, and astrocytes participate in Glial Fibrillary Acidic Protein (GFAP) regulation and neuroinflammation (Olmos et al., 1994; Siemian et al., 2018). Astrocytosis can be imaged by PET using [^11^C]-Deuterium-Deprenyl (Fowler et al., 1987), a radiopharmaceutical that targets monoamine oxidase type B (MAOB), or by the imidazoline-2 receptor radioligand [^11^C]-2-(4,5-dihydro-1H-imidazol-2-yl)-1-methyl-1H-indole ([^11^C]BU99008) (Tyacke et al., 2012, 2018). The former has been used to study astrocytosis in the cortex of patients with Alzheimer’s disease (Rodriguez-Vieitez et al., 2016) but its use in PD is limited by the high MAOB signal in the basal ganglia. [^11^C]-BU99008 has been validated in humans, showing a favorable kinetics profile. Despite the interest in astrocytosis, no PET studies in PD have been performed yet. On the other end, TSPO radiopharmaceuticals can bind both microglia and astrocyte cells. For this reason, the involvement of astrocytosis in the PET signal cannot be excluded.

In conclusion, TSPO imaging studies in patients are inconclusive in confirming the role of neuroinflammation in PD-associated neurodegeneration. Moreover, these studies cannot explain whether neuroinflammation is an early event since they have been performed only after the onset of clinical signs, i.e., when the neurodegeneration process is in an advanced stage. Another point regarding *in vivo* imaging of microglia and their role in disease progression is represented by the lack of radiopharmaceuticals able to discriminate between the different cell phenotypes. Despite several efforts, no radiopharmaceuticals with optimal biological and kinetics properties have been developed so far, to be used to image subtype-specific microglial phenotypes *in vivo*.

### PET Imaging of Neuroinflammation in Animal Models

Some studies have linked the presence of α-syn aggregates to inflammation in PD pathogenesis (Kim et al., 2013). Watson et al. (2012) showed that mice overexpressing human α-syn under the Thy promoter exhibited early focal microglia activation limited to the nigro-striatal circuit, before the appearance of DA neuron loss. According to this study, α-syn aggregates induce the overexpression of the TRL receptor family on microglia membranes, promoting microglia activation with the consequent release of TNF-α in the striatum. Microglia change their phenotype from resting to activated, first in the striatum (at 1 month of age), then in SN (at 5–6 months), finally in the cerebral cortex (at 14 months). Similar results were obtained using a transgenic rat model of PD overexpressing human native α-syn (Krashia et al., 2019). Rats overexpressing α-syn showed an increase of ionized calcium-binding adapter molecule 1 (Iba-1) positive cells in SN, striatum and dorsal hippocampus at 4 months of age, which was paralleled by an age-dependent increase of IFN-γ and decrease of the anti-inflammatory IL-10 in cerebrospinal fluid (CSF). Moreover, incubation of striatal slices of 2-month-old α-syn-overexpressing rats with INF-γ significantly reduced DA release, suggesting a cytokine role in accelerating dopaminergic transmission deficits. At this time, the basal locomotor activity was normal. Altogether, these studies suggest that α-syn deposition activates microglia leading to a shift to the proinflammatory pattern and a reduction in DA levels or a change in the electrophysiological properties of DA neurons, before motor signs appear. Studies performed in animal models indicated that the kinetic and distribution of the inflammatory responses are region and time-dependent, being limited to nigro-striatal regions first and spreading to the cortex later on.

Only a few studies have been conducted to investigate neuroinflammation in animal models of PD using *in vivo* imaging. The majority of the studies have been performed in acute neurotoxic models, i.e., in MPTP treated mice and 6-OHDA hemilesioned rats. MPTP induces oxidative stress and mitochondrial dysfunction particularly in dopaminergic neurons (Jackson-Lewis and Przedborski, 2007). This highly lipophilic pro-toxin is converted into the active form 1-methyl-4-phenylpyridinium (MPP+) by MAOB in astrocytes, then released in the extracellular space. MPP+ is actively transported inside dopaminergic neurons, where it blocks mitochondrial complex I (MC-I) causing neuronal death. MPTP also induces microglia activation (an increase of CD68-positive cells), cyclooxygenase-2 (COX-2) mediated oxidative stress (Teismann, 2012), increased IL-1α and IL-1β immunoreactivity in the olfactory bulb and striatum (Vroon et al., 2007), and α-syn nitration in the striatum and midbrain (Przedborski et al., 2001). MPTP can be administered under acute, sub-acute, or chronic protocols to induce a rapid or more progressive cell death, and different patterns of neuropathological changes. Different preclinical studies investigated the time course of nigro-striatal DA neuron degeneration in rodents and non-human primates treated with MPTP, using different PET radioligands (Ballanger et al., 2016; Kanazawa et al., 2017; Seo et al., 2019). To the best of our knowledge, only one study assessed neuroinflammation using PET (Belloli et al., 2017). In this study, [^11^C]-PK11195 was applied for the *ex vivo* monitoring of microglia activation to evaluate the effect of genetic deletion (KO mice) of the microglial regulator TREM2. Data showed a progressive increase of tracer binding in striatum and SN of mice acutely treated with MPTP (20 mg/Kg i.p. x 4, every 90 min), starting from day 2 after treatment. This was accompanied by the early and transient rise of TNF-α, Galectin-3 (Gal-3) and Iba-1, and a massive increase of TREM2, IL-1β, and TSPO transcript levels, which was paralleled by a progressive decrease of DAT, measured with the radioligand [^11^C]-N-(2-fluoroethyl)-2 beta-carbomethoxy-3 beta-(4-iodophenyl)nortropane [^11^C]-FE-CIT PET. These findings clearly indicated that activation of microglia is an early event after MPTP administration, and is even more anticipated in TREM2 KO mice. The acute MPTP model has, however, several limitations such as high general toxicity, acute dopaminergic cell death (within a few days), absence of α-syn aggregates, and inconsistent motor phenotype, thus it does not recapitulate the main neuropathological/clinical features of the disease and its progressive nature. No imaging study has been published on microglia/neuroinflammation in the more progressive subacute/chronic MPTP models. Oxidative stress was imaged in the unilateral 6-OHDA model, which is in most cases implemented in the rat. 6-OHDA is unable to cross BBB and is thus injected stereotactically into the medial forebrain bundle (mfb), SN, or striatum. 6-OHDA exerts its toxic effect through MC-I blockade and free radicals production (Betarbet et al., 2002). However, as for MPTP, 6-OHDA does not reproduce clinical and pathological features of PD, even when administered into the striatum to induce a more progressive death of SN neurons compared to mfb or SN injections (Przedborski et al., 1995). In the 6-OHDA hemilesioned rat, microglia activation was evaluated *in vivo* by PET and [^11^C]-PK11195 and the results compared with post-mortem immunohistochemistry (IHC) at different time points (Cicchetti et al., 2002). Anti-CD11b/c monoclonal antibody OX-42 staining showed high microglia/macrophages positivity in the striatum and SN of the lesioned hemisphere, starting from day 3 and persisting for 4 weeks after lesion. After 3 weeks, [^11^C]-PK11195 uptake resulted significantly increased in the striatum, SN and whole mesencephalon on the lesioned side. This was paralleled by a 65% reduction in DAT measured with the radioligand [^11^C]-CFT. At the same time point after striatal 6-OHDA injection, Venneti et al. (2007) compared [^11^C]-PK11195 binding with that of the new TSPO-specific compound [^11^C]-N-(2,5-Dimethoxybenzyl)-N-(5-fluoro-2-phenoxyphenyl)acetamide [^11^C]-DAA1106. A greater binding of [^11^C]-DAA1106 to damaged area was found, along with a modest increase of CD68 staining in the DA-depleted striatum *ex vivo*. Nevertheless, the IHC analysis was limited to the protein CD68 that is considered a marker of phagocytosis (Janda et al., 2018). In a recent study, 6-OHDA hemilesioned rats were evaluated weekly for 3 weeks after lesion with the new TSPO specific ligand [^18^F]-2-(4-fluoro-2-(p-tolyloxy)phenyl)-1,2-dihydroisoquinolin-3(4H)-one [^18^F]-FTPQ (Wu et al., 2019). Radiotracer uptake, measured as ipsilateral hemisphere to cerebellum ratio, progressively increased compared to the sham-operated hemisphere, reaching a maximum value at the 3rd week. After this time point, animals were sacrificed and brains evaluated for CD68 IHC revealing intense CD68 staining in the toxin-injected (i.e., dopamine-depleted) side.

A chronic model of PD has been generated in the mouse by repeated (up to four) injections of prostaglandin J2 (PGJ2) in SN, leading to gradual microglia activation and progressive DA neuronal loss in the nigro-striatal pathway (Shivers et al., 2014). The mechanisms by which PGJ2 activates microglia are yet unknown but IL-1 might be involved. The *in vivo* [^11^C]-PK11195 PET study was performed 1 week after the last of the four PGJ2 injections, showing a modest and not significant increase of tracer uptake, and a progressive increase of Iba-1 staining along with the number of PGJ2 injections.

Despite the studies using TSPO radioligands in toxin-induced PD models demonstrated the feasibility of using PET to monitor brain inflammation, several issues remain unaddressed, such as the identification of the timing of microglia activation relative to neurodegeneration, the microglial phenotype or the involvement of astrocyte, which is another target of TSPO radiopharmaceuticals. This is likely because the majority of these studies were not designed to investigate these aspects. Another limitation is represented by the use of acute models that do not reproduce the slow evolution of PD. Transgenic models could provide more information about disease onset and progression or the role exerted by misfolded proteins, as α-syn in synaptic demise. Indeed, transgenic mice overexpressing α-syn showed a loss of nigro-striatal projections, α-syn inclusions, and motor deficits (Betarbet et al., 2002). The same holds true for mice or rats where α-syn overexpression was achieved through the use of viral vectors (Klein et al., 2002; Song et al., 2015). No PET study has been done in these models yet, but an interesting work of Su et al. (2008) in transgenic mice overexpressing human wild type α-syn demonstrated the early enrolment of activated microglia. Moreover, the authors reported that in primary microglia-enriched cultures α-syn directly interacts with the CD36 scavenger receptor expressed on microglia cell membranes, triggering the expression of pro-inflammatory cytokines and ROS production. This might lead to dopaminergic neuronal death, synapsis toxicity, and propagation of the neuroinflammation/neurodegeneration process.

Another PD-associated gene is LRRK2. LRRK2 mutations are the most common pathogenic mutations associated with PD, and LRRK2 itself represents a risk factor for idiopathic PD (Reed et al., 2019).

LRRK2-associated PD is clinically and neuropathologically indistinguishable from idiopathic PD. LRRK2 is expressed not only in neurons but also in monocytes and microglia where it modulates a pro-inflammatory response (Moehle et al., 2012). Indeed, a recent study suggests that LRRK2 modulates microglia activity through the CX3C chemokine receptor 1 (CX3CR1) as revealed by experiments in LRRK2 null mice subjected to LPS administration (Ma et al., 2016). Interestingly, the response of LRRK2 KO cultured microglia to LPS resulted delayed or reduced, as after α-syn exposure, when compared to wild type cells (Russo et al., 2019), supporting a key role of LRRK2 in microglia regulation. A multi-tracer PET study was performed in rats overexpressing human LRRK2 G2019S (Walker et al., 2014). The authors compared 12-month-old LRRK2 G2019S rats to age-matched non-transgenic controls. PET assessed the density of DA with [^11^C]-methylphenidate ([^11^C]-MP), VMAT2 with [^11^C]-dihydrotetrabenazine ([^11^C]-DTBZ), and DA synthesis and storage with [^18^F]-FDOPA. The integrity of the dopaminergic system was also tested using [^11^C]-Raclopride ([^11^C]-RAC), a DA D_2_/D_3_ receptor antagonist, even in presence of methamphetamine displacement. Unfortunately, combined PET imaging did not evidence the dopaminergic alterations typical of PD showed in previous studies using mutant LRRK2 overexpressing rodents (Li et al., 2009; Dusonchet et al., 2011) or in presymptomatic subjects carrying LRRK2 mutation (Nandhagopal et al., 2008). This was probably due to the lack of a clear neurodegenerative phenotype in the LRRK2 G2019S model, even if authors used these animals as an early PD model. Further studies in elderly animals are needed to monitor disease progression and assess dopaminergic functionality and correlation with α-syn expression and inflammation over time to identify and characterize representative models of PD.

## PET Imaging of Mitochondria

Different post-mortem studies in PD patients have demonstrated deficits in the mitochondrial respiratory chain (Schapira et al., 1989; Sofic et al., 1992). Moreover, mutations or loss of function of proteins related to familial PD, like PINK1, Parkin, DJ-1 and α-syn, generate alterations in mitochondria metabolism or increased susceptibility to oxidative stress (Polymeropoulos et al., 1997; Puspita et al., 2017). The first evidence that mitochondrial dysfunctions are involved in PD pathogenesis emerged from MPTP studies. Whole transcriptome RNA deep sequencing in MPTP-treated mice showed deregulation in genes involved in DA synthesis and synaptic neurotransmission (Cheng et al., 2016). Moreover, treatment with the antioxidant compound [Cu^II^]- diacetyl-bis(N(4)-methylthiosemicarbazonato (Cu^II^(ATSM)), restored the expression of genes such as tyrosine hydroxylase, which controls DA biosynthesis and ROS generation, confirming that oxidative stress promotes dopaminergic dysfunction and that MPTP is a useful tool to investigate the role of oxidative stress in PD pathogenesis (Hung et al., 2012). The neuroprotective effect of the copper complex was confirmed using PET and the VMAT2 radioligand [^18^F]-AV133 (Hefti et al., 2010). The same complex of ATSM, labeled with [^64^Cu] or [^62^Cu] instead of non-radioactive copper, has been used to image mitochondrial electron chain function related to oxidative stress. In this study, patients injected with [^62^Cu]-ATSM showed an increase of striatal radioactivity uptake, which, according to the authors, might reflect an over-reduction state mainly caused by mitochondrial dysfunction, which leads to oxidative stress (Ikawa et al., 2011). Moreover, the radioligand uptake was found to positively correlate with disease severity and not with the age of patients. To the best of our knowledge, no study in animal models of PD neurodegeneration has been carried out using ATSM as radioligand, but *in vivo* imaging in oncological (Colombié et al., 2015) and ischemia (Huuskonen et al., 2017) models suggests that this compound might be a good candidate for both imaging and treatment of PD.

Another promising target to image mitochondrial activity in the living brain for application in neurodegenerative diseases is represented by MC-I (Tsukada et al., 2016; Kazami et al., 2019). The PET probe 2-tert-butyl-4-chloro-5-{6-[2-(2[^18^F]fluoroethoxy)-ethoxy]-pyridin-3-ylmethoxy}-2H-pyridazin-3-one ([^18^F]-BCPP-EF) was successfully evaluated as a radiopharmaceutical for the *in vivo* imaging of mitochondrial MC-I activity in brain and periphery in different animal models and conditions (Tsukada et al., 2014). The uptake of the MC-I inhibitor ([^18^F]-BCPP-EF) in cortical and basal ganglia regions significantly decreased in non-human primates chronically treated with MPTP. This decline correlated with a significant decrease of DAT ([^11^C]-β-CFT) and DA synthesis (β-[^11^C]-L-DOPA, [^18^F]-FDOPA) in the striatum, confirming the usefulness of this class of compounds in detecting mitochondrial dysfunction.

## PET Imaging of Other Neuronal Targets

α-syn aggregates in the brain and periphery represent an attractive target for detecting/monitoring PD. However, technical aspects limit the availability of α-syn specific PET ligands, especially the heterogeneity of fibrils and their posttranslational modifications, and the interference represented by the presence of beta-amyloid, to which these ligands also bind, in the brain of elderly PD patients. Researchers are working to overcome these issues (Helmich et al., 2018; Knudsen and Borghammer, 2018).

Different radioligands are available to study autonomic (sympathetic/parasympathetic) nervous system degeneration. Autonomic degeneration is particularly relevant in PD since it leads to gastro-intestinal dysfunctions or cardiac denervation responsible, among others, of constipation and orthostatic hypotension that are common in PD patients (Knudsen and Borghammer, 2018). To this purpose, [^11^C]-Donepezil PET was used in PD patients to measure significant AChE reduction in the small intestine of early PD patients, whereas [^18^F]-DA PET and [^125^I]-m-iodobenzylguanidine ([^125^I]-MIBG) scintigraphy were used to measure progressive heart denervation (Knudsen and Borghammer, 2018). These radioligands should be tested in animal models of PD, also to monitor autonomic changes in the presymptomatic phase.

Brain connectivity represents an additional target to study early modifications in PD. Together with nuclear imaging, functional and structural imaging using functional MRI (fMRI) could provide detailed information about abnormalities at the level of brain networks and connectivity (Helmich et al., 2018). fMRI revealed altered functional connectivity in cortical and subcortical regions, particularly in striatum, of PD patients (Hacker et al., 2012). These alterations occurred very early and were not associated with tremor manifestation, but varied depending on disease stage, PD subtype, and pharmacological treatment (Sreenivasan et al., 2019). The main problem of this application in rodents is represented by the small size of the mouse and rat brains. Then, although comparative MRI studies between PD patients and animal models, as the MPTP model (Hopes et al., 2016), have been performed to measure changes in regional volume and atrophy, alterations in connectivity or correlations with disease progression were not identified. In an interesting study, [^18^F]-FDG PET was applied to evaluate metabolic connectivity in 6-OHDA hemilesioned mice (Im et al., 2016). The voxel-wise-analysis was able to recognize disrupted connectivity in the cortico-striatal-thalamocortical loop, in particular, a decreased correlation between the right or left striatum and the visual cortex of DA-depleted animals, despite no alteration in regional metabolism was observed between the naïve and 6-OHDA groups. Additional studies comparing fMRI and [^18^F]-FDG PET in other animal models are needed to understand if these tools could help characterize early functional and metabolic changes in PD.

## Magnetic Resonance Imaging

MRI is applied in clinical practice to identify structural modifications in oncological and neurodegenerative diseases. In PD, most patients are addressed to MRI when motor symptoms have already developed, thus in an advanced disease stage, whereas imaging during the pre-motor phase is infrequent. Both conventional and non-conventional MRI plays an important role in the differential diagnosis between PD and parkinsonian syndromes (Baglieri et al., 2013). Conventional MRI, represented among others by T1- and T2-weighted imaging, proton density-weighted or diffusion-weighted (DWI) imaging, can determine the volume/size of affected areas, as SN, and iron content. Non-conventional MRI encompasses more recent techniques such as spectroscopy, diffusion tensor imaging (DTI), fMRI or morphometry MRI, that provide details of structural, functional and metabolic changes to improve diagnosis and treatment monitoring. Using conventional MRI sequences, Geng et al. (2006) measured the volume of SN and basal ganglia of PD patients at two different disease stages, and despite no changes occurred in total brain volume compared to controls, early PD patients exhibited a putamen volume reduction of 87.5%. Moreover, putamen atrophy inversely correlated with a clinical score, leading the authors to suggest a potential role of this marker in the diagnosis of early PD.

Morphometry MRI, a technique that allows the quantification of the sizes and shapes of brain structures, has been successfully applied to compare the volume of different brain areas and distinguish among PD, Multiple-System Atrophy (MSA) and Progressive Supranuclear Palsy (PSP) patients, with good sensitivity, specificity, and accuracy (Gama et al., 2010). In fact, at symptoms onset, PD can be confused with various atypical parkinsonian disorders, like the Parkinson variant of multiple system atrophy (MSA-P), or non-degenerative forms, including vascular parkinsonism. Interestingly, MSA is characterized by cytoplasmic inclusions of misfolded α-syn that lead to nigro-striatal degeneration accompanied by neuroinflammation, oxidative stress, and iron accumulation. MSA-P, PD, and DLB are classified as α-synucleinopathies, but unlike PD and DLB, MSA-P mainly affects oligodendrocytes, the most iron-rich cell population of the brain (Connor et al., 1990). Considering that iron, in its toxic Fe^2+^ form, seems to play a role in both translation and aggregation of α-syn, and that α-syn aggregation is accelerated in presence of DA and hydrogen peroxidase (Ostrerova-Golts et al., 2000), it is plausible to link iron concentrations to neuroinflammation, even if the mechanism is not fully understood. MRI is the easiest method to measure brain iron content *in vivo* using relaxation time of metal after magnetic field stimulation. The majority of the studies consider the R2* value (1/T2*) as the most appropriate to measure non-heme iron content. Hopes et al. (2016) evaluated 70 PD patients belonging to three different disease stages (*de novo*, early and advanced), and found R2* values to be significantly increased in the SN, putamen and caudate of *de novo* patients compared to control subjects. Moreover, R2* value was significantly increased in the SN of early and advanced patients compared to *de novo* patients, indicating that iron overload occurred faster in the first years of the disease, and did not linearly correlate with disease progression. In the same work, the authors found a positive correlation between MRI R2* value and iron overload in MPTP-treated mice. Also, You et al. (2015) found that increasing iron concentrations (measured by MRI R2*) with ferric ammonium citrate (FAC) exacerbated the damage induced by MPTP in murine SN, indicating that iron overload contributed to dopaminergic neuronal death. Moreover, the authors showed that elevating iron concentrations in SN also exacerbated oxidative stress promoting apoptosis and therefore contributing to PD onset.

Other authors applied Susceptibility-Weighted Imaging (SWI) MRI to estimate metal content in the brain, thus identifying typical patterns for PD, MSA-P, and PSP (Gupta et al., 2010). In MSA, iron typically accumulates in the putamen giving a greater signal reduction (hypo-intensity) compared to that observed in PD. Interestingly, Yoon et al. (2015) found that in MSA-P patients this hypo-intensity was also associated with the decrease of [^18^F]-FDG uptake measured with PET. In PD, increased iron concentration is found primarily in SN, but also in other regions like the putamen and globus pallidus (Kosta et al., 2006), and this seems to correlate with motor disability (Atasoy et al., 2004). Post-mortem analysis confirmed that iron deposits were particularly localized in macrophages, astrocytes, and reactive microglia of the SN and striopallidum of PD patients (Jellinger et al., 1990).

Using rat primary cultures and KO mice for the nicotinamide adenine dinucleotide phosphate oxidase 2 (NOX2) gene, which is involved in the generation of superoxide O_2_^−^, Zhang et al. (2014) investigated the role of microglia in dopaminergic neuron death induced by Fe^2+^.

They found that iron selectively decreased dopaminergic neuron viability *via* the activation of NOX2 in the microglia. Wang et al. (2013) demonstrated that iron homeostasis in dopaminergic neurons is influenced by pro-inflammatory cytokines released by the activated microglia. Specifically, the authors demonstrated that IL-1β and TNF-α can increase iron transport in primary cultures of ventral mesencephalic (VM) neurons.

Altogether, these data provide evidence that MRI can capture iron deposition and inflammation as potential markers of early PD and that anti-inflammatory and iron chelators, such as deferoxamine (DFA), might represent valuable therapeutic approaches in PD.

## Conclusions

PD is the second most common neurodegenerative disorder and is characterized by motor and non-motor symptoms that worsen as the disease progresses. Current treatments are symptomatic and predominantly focused on the rescue of defective dopaminergic transmission. The main risk factor of PD is represented by age, but genetic and environmental factors are emerging as important determinants. Several targets have been investigated in PD to better understand its pathogenesis, follow the progression of the disease and the effectiveness of treatments, and shed light on the early events that precede clinical onset. Neuroinflammation might represent a very promising marker since it represents an early event and is involved both in disease onset and progression. In this respect, preclinical longitudinal studies in selected animal models could help clarify whether neuroinflammation precedes or follows neuronal demise. Up to now, PET imaging with specific radioligands has been applied to the diagnosis and monitoring of disease progression in patients, but only after symptoms onset. Moreover, no studies correlated the TSPO signal with the specific neuroinflammatory phenotype, even if they clearly demonstrated the feasibility of using PET to monitor brain inflammation in animal models of PD. Unfortunately, this technique cannot discriminate between resident and peripheral inflammation without using combined *in vitro* analysis.

With the limitations above, *in vivo* imaging of neuroinflammation, better if combined with traditional molecular analysis, allows to understand the preclinical modifications occurring in PD. However, this might have translational potential and an impact on the clinical practice only if used in predictive animal models.

## Author Contributions

All authors selected the related literature, conceptualized, designed, and wrote the manuscript. All authors edited and revised the manuscript.

## Conflict of Interest

The authors declare that the research was conducted in the absence of any commercial or financial relationships that could be construed as a potential conflict of interest.
